# Industrially Produced Plant-Based Food Products: Nutritional Value and Degree of Processing

**DOI:** 10.3390/foods13111752

**Published:** 2024-06-03

**Authors:** Marta Maganinho, Carla Almeida, Patrícia Padrão

**Affiliations:** 1 Faculdade de Ciências, Universidade do Porto, 4169-007 Porto, Portugal; 2 Faculdade de Ciências da Nutrição e Alimentação, Universidade do Porto, 4150-180 Porto, Portugal; 3 EPIUnit—Instituto de Saúde Pública, Universidade do Porto, 4050-600 Porto, Portugal; 4 Laboratório para a Investigação Integrativa e Translacional em Saúde Populacional (ITR), 4050-600 Porto, Portugal

**Keywords:** plant-based food products, nutritional quality, ultra-processed foods, NOVA

## Abstract

The plant-based food market is rapidly growing, offering innovative options to meet consumer expectations. However, a comprehensive analysis of the nutritional quality of these foods is lacking. We aimed to characterize industrial plant-based food products’ nutritional value and degree of processing. A cross-sectional study was conducted on two market-leading Portuguese food retail chains by assessing the nutritional composition of all the available pre-packaged plant-based food products (*n* = 407). These products were categorized into meal alternatives, dairy alternatives, and other products containing dairy/meat alternative ingredients including ready meals and desserts. The products’ nutritional quality was assessed according to the cut-offs established by the Portuguese Directorate General of Health [DGS] on total fat, saturated fat, sugar, and salt, and considering the degree of processing using NOVA classification. One-tenth of the products were classified as having a high total fat, saturated fat, sugars, or salt content. In some sub-categories, half of foods were classified as high in saturated fat, and over two-thirds were considered high salt products. Less than one-third exhibit a good nutritional profile based on the national cut-offs. A total of 84.3% of plant-based food products were ultra-processed. These findings emphasize the need to improve the nutritional profile of plant-based options.

## 1. Introduction

The last few years have been marked by an exponential increase in the demand and consumption of plant-based food products [1]. This trend is driven by the widespread promotion of plant-based diets, the reduced intake of animal-source foods, and the limitation of highly processed foods consumption. These dietary changes offer co-benefits related to human health improvements and reduced environmental impact [2,3,4,5]. Health, environmental, and animal welfare reasons are among the main consumption determinants, although the desire for new food experiences and especially social influence have been huge drivers to the rising trend of plant-based diets [1,6,7,8]. Consequently, a simple increase in the availability of plant-based food products can act as a cue leading to a food behavior change [9].

In this context, the plant-based segment market has differentiated positively over time, with a food industry strongly focused on innovation and the development of new products, to meet the expectations and needs of the modern consumer [10,11,12]. Indeed, the sales of plant-based options in Europe increased by 21% from 2020 to 2022 [13], and according to data from the Plant-Based Food Association [14], the 2022 U.S. plant-based market reached USD 8.0 billion, representing an impressive 6.6% growth of retail sales, reflected in a total of 44.5% three-year growth. Despite the pandemic challenges and the constraints in the food supply chain followed by rising inflation, the plant-based trend has a staying power. It will continue to drive growth in the food industry, with a global market valuation of USD 11.3 billion in 2023 and a forecasted USD 35.9 billion for 2032 [15]. These values result from a growing number of flexitarians, consumers who have effectively contributed the most to the increase in sales of plant-based products and encouraging continuous innovation in this market segment [16].

The increasingly diversified range of products available on the market (e.g., meat alternatives, dairy alternatives, plant-based ready meals), facilitates the adherence to lower/animal-based-free diets and are commonly perceived as healthier options [17,18,19]. However, insufficient attention to the quality of plant-based food products has been given [20] an issue of concern, as recent evidence has been pointing out that these products might be predominantly ultra-processed with high content of fat, sugars, and salt [19,21]. The increased availability and intensive marketing of ultra-processed foods (UPFs), frequently characterized by being energy-dense, nutrient-poor food products, highly convenient, and hyper-palatable, leads to the increased consumption of these foods, which has been recognized as a key dietary risk factor for diet-related non-communicable diseases (NCDs), such as overweight and obesity [22,23]. The current range of plant-based food products contributes to an oversimplified perspective regarding plant-based diets, as they can often be perceived as practical alternatives [10,24]. However, according to the NOVA classification system [25], many of the plant-based food products could be classified as UPF products, typically characterized by high extent of processing, the use of starches, protein isolates, sugars, fats, and frequent addition of colors and flavors to improve sensory appearance, and emulsifiers and other cosmetic additives to promote a higher shelf-life [25,26]. Nevertheless, there is an important knowledge gap regarding the composition of the plant-based options launched daily on the market.

Although plant-based diets are reported to have health benefits, their quality may be heterogeneous and should be carefully assessed [26]. The present study aims to characterize the nutritional quality of pre-packaged plant-based food products sold in the Portuguese market by assessing their nutritional composition and degree of processing.

## 2. Materials and Methods

A cross-sectional study was conducted to collect labeling data on the plant-based food product supply of the two main Portuguese food retail chains.

The nutritional information of the plant-based food products of every brand available for sale was collected using a systematic approach and compiled in a database. All pre-packaged products with plant-based mentions on the label were included, considering the following inclusion expressions: plant-based, vegan, vegetarian, meat-free, meatless, dairy-free, non-dairy, and also expressions such as “made from plants” and “veggie-based”. Subsequently, the list of ingredients for each eligible product was consulted, and those containing ingredients of animal origin were excluded.

(a) Data collection

Product information was collected from two representative supermarkets regarding availability (one for each food retailer) from April 2021 to November 2022. The ingredient list of the products was analyzed to guarantee that only food products exclusively plant-based were included, e.g., made of ingredients from plants and not containing animal ingredients of any kind (including milk, egg protein, or egg white). The research team went to the referred supermarkets and collected information from the food packaging of the eligible foods using photographs regarding the ingredients list and nutritional composition by 100 g/mL (energy (kcal), total fat (g), saturated fat (g), carbohydrates (g), sugars (g), protein (g), fiber (g), and salt (g)), mandatory parameters according to the Council Regulation (EU) 1169/2011 [27].

 (i) Plant-based food product categorization

The eligible food products were grouped according to the definition proposed by Beacom et al. (2021) [11] into the following plant-based food categories: 1. meat alternatives (e.g., meat-free burgers, sausages, nuggets, or tofu); 2. dairy alternatives (e.g., plant-based beverages, yogurts, or cheeses manufactured from ingredients such as soya, coconut, rice, or almond); and 3. Other products that contain a dairy or meat alternative ingredient, such as plant-based desserts and ready meals. 

Considering the diversity of products among the different plant-based food categories, sub-categories were additionally established based on the products’ main ingredients and/or typology (Table 1).

The sub-category “mixed” was applied to any food product of dairy alternative categories, made from a mixture of plant-based food matrices (e.g., soy and almond; or other combination).

 (ii) Nutritional composition

The nutritional data of the eligible products, including energy value, macronutrients (total fat, saturated fat, carbohydrate, sugars, protein, fiber), and salt were, respectively, reported in kJ/kcal and grams per 100 grams of the product. In addition, plant-based foods of different categories were classified as having low, medium, or high energy value (kcal/100 g) and content (g/100 g) of saturated fat, sugar, and salt, using the label decoder proposed by the National Program for the Promotion of Healthy Eating (PNPAS) that establish cut-offs for different nutritional parameters [28], according to the Nutrient Profiling Model recommendations of the UK Department of Health [29]. Food categories were ranked based on the percentage of products observed in each of these levels.

 (iii) Degree of Processing

All the food products assessed were classified according to the NOVA food classification system [25], which divides foods according to the degree of processing. Groups 1 and 2 are the classifications applied to foods with a lower degree of processing, related to unprocessed or minimally processed foods (e.g., fresh fruits, vegetables, and grains) and processed culinary ingredients such as oils and sugar, respectively. Group 3 comprises foods with a moderate processing degree, while Group 4 represents the higher degree of processing associated with ultra-processed foods, which are industrial formulations typically high in additives and with a high extent of processing. Considering the characteristics of the eligible products included in this study, the items were only classified into minimally processed, processed, or ultra-processed foods, groups 1, 3, and 4 of the NOVA classification system, respectively. 

Following the NOVA guidelines, we identified UPFs within the selected plant-based food product samples by examining ingredient lists. Additionally, a detailed analysis of the ingredient profile of ultra-processed plant-based products was conducted by counting the ingredients that contributed to classifying each food as ultra-processed.

(b) Data Analysis

Descriptive statistics (median and interquartile range) of energy, macronutrients, and salt content were used to describe the nutritional composition of the eligible food products. The normality of data distribution was first assessed through the Kolmogorov–Smirnov test. Kruskal–Wallis and Mann–Whitney tests were applied as appropriate to test the variability in energy value and nutrient content of the products according to sub-categories. The IBM SPSS statistics version 29.0 was used for the statistical analysis. The significance level was set at *p* ≤ 0.05.

## 3. Results

Of a total of 543 plant-based food products identified with eligible plant-based mentions on the label, 136 were excluded because they contained ingredients of animal origin, resulting in a total of 407 products for the analysis. In terms of plant-based food categories’ representation, dairy alternatives lead the offer in this market segment, with 55.0% of the products collected corresponding to this category, especially plant-based beverages (29.6%), followed by meat alternatives (30.0%) (mainly, plant-based burgers (30.0%)) and other plant-based options (15.0%), such as ready meals (40.0%) and desserts (60.0%), among which plant-based frozen meals (75.0%) and plant-based ice creams (52.7%) are the predominant options, respectively.

### 3.1. Nutritional Composition of Plant-Based Food Products

The nutritional composition of plant-based food products by category is described in Table 2. The three categories assessed cover a wide range of plant-based food products with heterogeneous nutritional compositions. The meat alternative category has the highest energy content and higher levels of total fat, fiber, protein, and salt compared to the dairy alternatives and others’ categories, which stand out for their carbohydrate and sugar content.

The following subsections present the nutritional composition results by category.

#### 3.1.1. Meat Alternatives

The median energy value of meat alternatives ranged between 146 kcal/100 g in tofu and seitan alternatives and 250 kcal/100 g in nuggets (Table 3). 

The total fat content ranged between 8.1 g/100 g in tofu and seitan and 18.0 g/100 g in sausages, with the others’ category presenting the lowest saturated fat level (0.8 g/100 g) and sausages the highest (2.4 g/100 g). The total carbohydrates ranged between 0.6 g/100 g in sausages and 20.0 g/100 g in nuggets. The lowest median sugar content was observed in tofu and seitan category (0.5 g/100 g) in opposition to falafel which presented the highest sugar median (2.8 g/100 g). In terms of the fiber median content, it ranged between 0.5 g/100 g in sausages and 5.9 g/100 g in the falafel category. About the protein content, the falafel category presented the lowest median value, while meatballs and tofu and seitan had the highest median content (6.7 g/100 g vs. 16.0 g/100 g and 16.5 g/100 g, respectively). Significant statistical differences were observed for all the nutrients across products.

#### 3.1.2. Dairy Alternatives

The nutritional composition analysis of this category is presented according to the sub-categories defined, i.e., plant-based beverages, yogurts, and cheeses (Table 4, Table 5, and Table 6, respectively). 

Regarding plant-based beverages, the median energy value ranged between 26 kcal/100 mL and 54 kcal/100 mL in almond and rice beverages, respectively. Rice beverages were the ones that presented the lowest median total fat content (1.0 g/100 mL), and soy beverages had the highest (1.8 g/100 mL), with a similar scenario observed for saturated fat content. The total carbohydrates and sugar content ranged between 2.5 g/100 mL and 2.1 g/100 mL and 11.0 g/100 mL and 4.8 g/100 mL in “others” and rice beverages, respectively. Regarding fiber, the lowest median was observed in rice beverages (0.3 g/100 mL) and the highest in oat beverages (0.8 g/100 mL). The median protein content ranged between 0.2 g/100 mL and 3.0 g/100 mL in rice and soy beverages, respectively. Moreover, considering the plain and flavored beverages, it was possible to observe significant differences in the nutritional composition, with the flavored ones standing out not only with higher median energy but mainly with higher total carbohydrates and sugar median quantities (Table 4). Statistical differences in the nutritional content were observed among dairy alternatives categories, except for salt.

Regarding plant-based yogurts, statistical differences in nutritional content were observed among its sub-categories. The median energy value of the plant-based yogurts ranged between 56 kcal/100 g and 90 kcal/100 g in the “mixed” and coconut yogurts, respectively. Regarding the total fat content, the soy yogurts were the ones that presented the lowest median (2.1 g/100 g) and the almond ones the highest (4.7 g/100 g), with the coconut yogurts exhibiting the highest saturated fat content, 3.7 g/100 g in comparison with the other yogurt options (0.4 g/100 g in the soy and almond yogurts; 1.1 g/100 g in the “mixed” ones). The total carbohydrates varied between 2.4 g/100 g and 11.8 g/100 g in the “mixed” and coconut yogurts. Nevertheless, the almond yogurts had the lowest median sugar levels (0.8 g/100 g), and soy had the highest (7.7 g/100 g). Regarding fiber content, the almond yogurts presented the highest median value, 4.5 g/100 g, while the remaining alternatives showed levels between 0.64 and 1.0 g/100 g. The protein content was significantly lower in the coconut and almond yogurts compared to the soy and mixed options (0.5 g/100 g and 2.0 g/100 g vs. 3.7 g/100 g and 3.9 g/100 g, respectively). Considering the different yogurt typologies, the median energy value ranged between 55 and 84 kcal/100 g in natural and Greek, respectively. The flavored and Greek yogurts have the highest carbohydrate and sugar content, while the natural yogurts have the lowest sugar levels (Table 5). 

Regarding plant-based cheeses, the median energy value was 281 (260–292) kcal/100 g, and no statistical differences were found for energy and macronutrients among sub-classifications and typologies (Table 6).

#### 3.1.3. Ready Meals and Desserts

The nutritional composition analysis of this category was presented according to the sub-categories defined, i.e., ready meals and desserts, with the median results expressed in Table 7.

Considering the plant-based ready meals, the canned options presented the lowest median energy value (76 kcal/100 g) and the refrigerated ones had the highest levels (161 kcal/100 g). The total fat content ranged between 2.7 g/100 g and 8.9 g/100 g in the frozen and refrigerated ready meals, respectively, with a similar pattern for saturated fat. Regarding the total carbohydrates, the canned ready meals presented the lowest median (4.0 g/100 g) in opposition to the refrigerated ready meals which showed the highest median (16.0 g/100 g). However, in terms of sugar contribution, it ranged between 1.8 g/100 g and 3.1 g/100 g in the refrigerated and canned ready meals, respectively. For the fiber content, the median was significantly lowest in the refrigerated ready meals (1.1 g/100 g) and highest in the frozen ones (3.2 g/100 g). In terms of protein, the median ranged between 3.6 g/100 g and 5.7 g/100 g in the refrigerated and canned plant-based meals. Regarding the salt content, the lowest median was observed in the frozen meals (0.7 g/100 g) and the highest in canned (1.0 g/100 g). 

Regarding plant-based desserts, the median energy value was significantly higher in ice cream compared to creamy desserts, with a median energy value of 250 kcal/100 g and 95 kcal/100 g, respectively. Regarding the median fat and sugar content, the creamy desserts exhibited 2.3 g/100 g of fat (of which 0.6 g/100 g was saturated fat) and 11.0 g/100 g of sugars in comparison to the ice creams, which showed 13.9 g/100 g of fat (of which 11.0 g/100 g was saturated fat) and 21.2 g/100 g of sugars.

### 3.2. Nutritional Profile of Plant-Based Food Products

According to the food label decoder of PNPAS, the nutrition profile by food category is expressed in Table 8. A total of 29.0% of the plant-based food products presented a nutrition profile characterized by a low level of all the target nutrients (total fat, saturated fat, sugars, and salt). Overall, the proportions of products classified as having high total fat, saturated fat, sugars, and salt were 5.9%, 7.4%, 3.2%, and 10.3%, respectively. Cheese and meat alternatives reached a proportion of 71.4% and 29.5% of high salt products, respectively, while a high content of saturated fat was observed in 50.0% of the desserts. The remaining products varied in the cut-off levels, with no products revealing high levels for the four nutrient categories, with only one product showing high levels in three of them.

### 3.3. Degree of Processing of Plant-Based Food Products

Considering the NOVA classification system, 84.3% of the plant-based food products collected were classified as ultra-processed, followed by 15.5% of the processed foods and 0.2% corresponding to a single product classified as a minimally processed food product in Table 8. Table 9 presents the total number of ingredients responsible for the ultra-processed classification (NOVA group 4).

It can be observed that the median (P25–P75) total number of ingredients included in a plant-based food product is 15 (10–18), from which a median of 4 (2–5) ingredients were responsible for the ultra-processed classification. Nuggets and meatballs were the meat alternatives with the longest list of ingredients (22 (21–23) and 20 (17–23)), respectively, and those with the most ingredients which classified as ultra-processed (6 (6–7) and 8 (7–9); *p* < 0.01). Regarding dairy alternatives, rice and oat beverages presented simultaneously the shortest ingredient list and the ones with fewer ingredients, which classified them as ultra-processed (6 (4–10)–8 (5–11); *p* < 0.01 and 1 (0–2); *p* < 0.05, respectively). The main NOVA group 4 ingredients responsible for classifying ultra-processed plant-based food products are exhibited in Table 10.

## 4. Discussion

Overall, and considering the results of the present study, the current Portuguese plant-based food offer includes a range of products with high energy value, total fat, sugar, salt, and low protein content. Most plant-based food products were classified as ultra-processed, presenting extensive ingredient lists with a high set of associated ultra-processed ingredients. In addition, less than one-third exhibit a good nutritional profile based on the national cut-offs, i.e., low levels of total fat, saturated fat, sugar, and salt. 

In fact, the World Health Organization has raised attention regarding the urgency of evaluating the nutritional profile of these plant-based food alternatives since most of them were expected to be ultra-processed products [19,20], recognized for their high-energy density, and frequent consumption of which may be positively related to a higher risk of non-communicable diseases (NCDs) [30,31,32,33].

This fact assumes special relevance given the recognized growth potential of the plant-based food options available on the shelves, despite already being considerable, as demonstrated by the number of items collected in this study—407 products. These findings not only show the commitment of food retailers to expand and diversify their offers but also signal the growing interest of Portuguese consumers in plant-based alternatives. 

Regarding specifically dairy alternatives, this is an increasingly pronounced segment of the plant-based market [34], with growing investments to expand its portfolio (e.g., plant-based beverages, plant-based yogurts, and cheese analogs) in order to meet current consumer demands. In fact, dairy alternatives were the predominant plant-based food products found on supermarket shelves and assessed in the present study (55%), with plant-based beverages leading this segment (64.4%), followed by plant-based yogurts (32.4%), and cheese analogs (3.1%) that are dairy alternatives for which exploration and innovation have been increasing. Particularly, plant-based beverage consumption has become a popular trend, as options like oat, almond, rice, or soy, are appealing alternatives to dairy milk and simultaneously associated with lower environmental impact and larger health benefits (e.g., frequently richer in vitamins and minerals and cholesterol and lactose-free) [35,36,37,38]. Additionally, vegan, vegetarian, and flexitarian consumers, especially health-conscious individuals, and the rising prevalence of lactose intolerance worldwide have been key drivers of the increased value of this plant-based segment, which is also held by the countries where dairy milk consumption is culturally lower [35,36,38].

Considering plant-based beverages, our findings revealed some variability among the types of beverages. Regarding the nutritional values observed in the present study and comparing to the Italian market [39], we can note similar results, with oat (7.7 g/100 g vs. 7.9 g/100 g) and rice (11.0 g/100 g vs. 12.0 g/100 g) beverages being the rich options in total carbohydrates, with rice ones standing out for their higher sugar levels (4.8 g/100 g vs. 6.2 g/100 g) and soy beverages for the highest protein content (3.3 g/100 g vs. 3.0 g/100 g). In the Craig and Fresán (2021) [40] study, 55.0% of the beverages had less than 5.0 g/serving of sugars compared to the 73.1% of the present study. Furthermore, if considering the cut-offs proposed by the Portuguese Directorate General of Health [Direção-Geral da Saúde (DGS)] for beverages (g/100 mL) [28], only 31.7% of the assessed beverages satisfy the recommended low levels of sugars (≤2.5 g/100 mL) in opposition to the 68.3% of plant-based beverages that presented medium level of sugars (2.5–11.25 g/100 mL). Moreover, we found that flavored beverages (e.g., vanilla, coffee, and chocolate), compared to plain ones, have about twice the sugar content, in line with data that indicate a tendency for this type of beverage to be sweeter [40]. In terms of protein content, soy beverages presented the highest levels (3.0 g/100 mL), similar to findings relative to the soy-based beverages of New Zealand [41] and also in the European Market [39,42], 2.87 g/100 g and 3.0–3.4 g/100 g, respectively, which also shows that the remaining plant-based beverages options have a much lower protein content of less than 1.0 g/100 mL. Regarding fat content, and especially saturated fat levels, and according to Clegg et al. (2021) [43], the plant-based beverages analyzed presented ≤ 0.3 g/100 ml, and therefore were also in accordance with the less restricted cut-off recommended by the DGS (≤0.75 g/100 mL). Recently, Drewnowski et al. (2021) [44] proposed nutrient standards for milk alternatives, suggesting a maximum energy value of 85–100 kcal/100 g, not less than 2.2 g/100 g of high-quality protein, saturated fat content lower than 0.75 g/100 g, and 5.3–6.25 g/100 g of added sugars. Attending to the proposed values, only 21.4% of the plant-based beverages included in this study accomplished those recommended nutrient levels.

Looking closely at plant-based yogurts, our results revealed that the soy-based yogurts, besides being the main plant-based option available, are the ones with lower energy value (68 kcal/100 g) in opposition to the nuts-based (e.g., almond) yogurts (77 kcal/100 g), and the coconut yogurts (90 kcal) that are the most caloric options, in accordance with the results presented by Clegg et al. (2021) [43], i.e., 68.4 kcal/100 g, 96.8 kcal/100 g, and 111.7 kcal/100 g, respectively. In line with our results, the coconut and nuts yogurts (e.g., almond), due to their fatty food matrix, are the products that most contribute to the higher fat content of the plant-based yogurts [43,45,46]. Nevertheless, in terms of saturated fat, plant-based yogurts have low levels except for those that are coconut-based [47]. Regarding the protein content, evidence shows relatively lower levels than in dairy yogurts, with an overall picture for the plant-based yogurts’ protein similar to our findings, i.e., of about 3.6 g/100 g, with soy-based as the ones with the highest content [43,45,47,48]. Despite the launch of non-dairy protein yogurt options, it is relevant to highlight that these cannot be automatically considered rich in protein if it does not meet the minimum 12% of the total energy value (VET) established by the Regulation (EC) No 1924/2006 [49]. It is fully recognized that the role of yogurt as a nutrient-dense food capable of providing multiple health benefits, in virtue of its composition in probiotic bacteria and/or prebiotic compounds, significantly contributes to improving gastrointestinal health and reducing the risk of chronic diseases, such type II diabetes, and breast or colorectal cancer [50,51,52]. However, some concerns have been raised regarding the possibility of it being unrecognized as a high-sugar food source [48,53], in line with the results of the present study (i.e., 6.8 (2.2–8.2) g/100g) corroborated by Moore et al. (2018) [48], who reported that dairy alternatives to yogurt available in the UK market presented about 9.2 g/100 g of sugars. Evidence shows that compared to dairy ones, the plant-based options have a higher energy value, total fat, and carbohydrate content, with similar levels of saturated fat, sugar, and salt [43,45].

Albeit the lack of evidence regarding the nutritional composition of plant-based cheeses, recent findings show that these dairy alternatives are energy-dense products with a high energy value, total fat, saturated fat, as well as high levels of salt [54,55]. Most cheese alternatives are made of coconut oil [19,45,54,55], a rich source of saturated fat. Furthermore, we observed that 71.4% of the plant-based cheeses presented salt levels greater than 1.5 g/100 g, similar to values reported by previous studies [19,54,55]. As these dairy alternatives are mainly coconut-oil based, the protein content is very limited (i.e., 0.0 (0.0–1.6) g/100 g) and, in some cases near-zero, in line with previous studies, 0.0 (0.0–0.3) g/100 g and 0.4 (0.0–0.6) g/100 g, respectively [54,55].

Recent findings revealed an increase in the global consumption of animal-source foods (e.g., unprocessed red meat, eggs, milk, processed meat, seafood, and cheese) [56]. It is important to consider that animal-source foods, especially meat, are among the food groups that most influence both human and planetary health [57,58]. The intense use of natural resources and the large proportion of greenhouse gas emissions (GHGEs) are environmental burdens related to the high animal consumption patterns that have been threatening the sustainability of the global food system [5,59,60].

Hence, in a time where the unsustainable food system calls for an urgent reduction in meat consumption, finding protein alternatives is a great challenge, and the innovation and development of meat alternative products have been an important strategy to help consumers shift to increasingly low meat/meat-free diets [61]. According to Bryngelsson et al. (2022) [62], meat alternatives are predominantly rich protein sources, with low saturated fat content and a source of fiber. The present study shows indeed a lower content of saturated fat and high fiber levels of meat analogs, almost five times lower and three times higher, respectively, in line with Tonheim et al. (2022) [63], who observed almost six times lower levels of saturated fat and four times higher fiber levels. Although plant-based meat references have similar fat levels to traditional meat products, the main differences in saturated fat content are due to the lipidic profile of the plant ingredients used on meat analogs, i.e., mostly mono- and polyunsaturated fat, with short-chain and intermediate-chain fatty acids’ introduction as the main differentiating factor [64]. The frequent use of cereals and pseudocereals in meat analog formulations leads to the highest fiber levels of these products, which, when consumed as part of a balanced diet, can contribute to increased fiber intake [63,65]. Despite this, and compared to meat references, not all alternatives provide optimum protein levels [66]. In fact, there has been observed a high variability of protein content among the remaining plant-based options, which is usually lower than the animal-based ones [19,67,68]. Moreover, despite the continuous improvement of meat alternatives and the increase in protein levels presented, it is important to highlight that plant-based options can not be considered equivalent to their animal-based versions, because, aside from the fact that they are multi-ingredient food products, the protein quality is different (i.e., distinct PDCAAs, presence of anti-nutritional factors, essential amino acids deficiencies) [69,70] and can be only claimed as a high source of protein if this corresponds to 12% of the total energy value (VET) [49]. Furthermore, in the present study, 59.0% and 29.5% of the meat alternatives exhibited medium (0.3–1.5 g/100 g) and high (≥1.5 g/100 g) levels of salt, respectively, in line with recent data from Spanish and Australian markets, which showed that meat analogs have upper-moderate to high salt levels [65,71], a dietary factor intimately related to the global burden of disease. The nutritional composition of meat alternatives, along with extensive ingredient lists composed of food components with a high degree of processing associated, leads to the ultra-processed classification of these plant-based options according to the NOVA classification system. In our study, 86% of the meat analogs were considered UPFs, in agreement with the 94% identified in the study of Rizzolo-Brime et al. (2023) [71].

The current fast-paced, dynamic lifestyles have driven consumers to demand more convenient food options, such as ready meals that can be an easy and timely alternative to the regular diet [72]. However, when compared to home-cooked meals, these food options are not just more expensive, but they also frequently present lower nutritional quality, as demonstrated by recent data that reveals that plant-based ready meals present high fat and salt content [73,74] in line with our results. Nevertheless, considering the lack of culinary skills and the unfamiliarity with replacing the best animal-based proteins [7], plant-based ready meals could facilitate the transition to plant-based diets.

It is indisputable that the growing market of plant-based food products and the increasing portfolio of several alternatives have responded to modern consumers’ demands, who increasingly seek and expect a more diversified plant-based diet [12,75,76]. However, the higher quality of this diet cannot be taken for granted when the innovation and development of new plant-based products are mostly associated with an ultra-processed offer, as observed in the present study. In fact, meat and dairy alternatives are the plant-based options that have contributed most to the increased consumption of ultra-processed products, leading not just to changes in diet nutritional adequacy but also to unhealthy plant-based diet indices [77,78]. In addition, it is well established that UPFs are frequently energy-dense foods, usually nutrient-depleted, and positively correlated with an increased intake of free sugars, sodium, total fat, and saturated fat [79,80]. Nevertheless, food processing has played a crucial role in society’s evolution by increasing the diversity of food products and ensuring food safety through extended shelf life and the control of health risk factors from foodborne diseases—a persistent global public health challenge [81]. Even so, the role of UPF consumption in the increased prevalence of NCDs, with a special contribution to the global obesity pandemic and increased risk of all-cause mortality is currently fully recognized [30,82,83]. The UPF consumption is significantly high worldwide, with recent data showed an energy contribution ranging from 14% to 44% across 22 European countries [84]. Consumption levels could be underestimated, as consumers still present some confusion regarding foods’ degree of processing identification, contributing to the uncertainty of food purchases [85]. Although the increasing availability of plant-based food products may facilitate the transition to a more plant-based diet, this food trend could also contribute to the perpetuation of excessive UPF consumption with negative health outcomes as well as to nullify the associated sustainability of plant-based diets [86]. However, little attention has been given to the impact that the growing consumption of ultra-processed plant-based food products might have in the long term.

Despite some of the disadvantages of food processing which is often associated with energy-dense and low nutrient profile, new and emerging technologies are continually being developed to enhance the integration and contribution of food processing in the food system [84]. In this context, we should take a cautious approach when studying nutritional adequacy and processing. The association is not absolute nor true for all cases.

### Strengths and Limitations

To the best of our knowledge, this is the first study aiming to comprehensively evaluate the nutritional quality of the plant-based food products offered by the leading Portuguese food retail market chains. We systematically collected nutritional data from a broad range of plant-based food products, assessing the nutritional quality of three plant food categories: meat alternatives, dairy alternatives, and “others” (i.e., plant-based ready meals and desserts). However, the fact that the collected data were derived only from plant-based products of two food retail chains could be a limitation of the study. Nevertheless, it is important to highlight that we consider the two major Portuguese supermarket retail chains, which account for approximately 50% to 75% of the market share [87]. Therefore, we believe that the present findings are not only an overview of the remaining Portuguese food retailers but may reflect a wider market as the main plant-based food brands are multinational. Furthermore, the fact that the degree of processing was assessed according to NOVA classification could be somewhat of a limitation as this system is based on a qualitative approach that brings ambiguity and differences in interpretation [88,89,90] that may underestimate the healthiness of some nutrient-dense foods, adding no value to existing nutrient profile systems [91]. However, new classification systems of processing degree evaluation, such as SIGA, intended to address NOVA limitations, suggest that foods with over five ingredients are highly likely to be UPFs [92], indicating that products with longer ingredient lists are more likely to contain multiple markers of ultra-processing (MUPs), supporting our study results. Nevertheless, and despite our findings being in line with recent evidence, future multidimensional assessment should be performed for a broader evaluation of the nutritional profile of plant-based food products and for a more accurate estimation of their long-term impact on health.

## 5. Conclusions

The plant-based food market presents a wide diversity of nutritional composition, although the proportion of products classified as having a high content of total fat, saturated fat, sugars, or salt reached one-tenth. In some sub-categories, half of the foods were classified as high in saturated fat, and over two-thirds were considered high salt products. Moreover, the present study’s findings revealed that most plant-based alternatives present extensive ingredient lists composed of ingredients with a high degree of processing, intimately responsible for more than 75% of the plant-food products classified as UFPs according to the NOVA classification system.

Despite using data on plant-based food products available from the two market-leading Portuguese food retail chains supermarkets, we believe that our results may reflect the European panorama since a large part of the food products available in Portugal are imported and belong to multinational companies.

Given the increasing growth of plant-based foods, we are convinced that the results of this study alert us to the contribution of ultra-processed foods to this food segment, which can potentially impact the quality of the diets of specific groups of the population, now and in the future.

The umbrella of plant-based food product categories covered in this study emphasizes the need to improve the nutritional profile of industrial plant-based food supply and nutritional literacy promotion among consumers to ensure a transition to plant-based diets based on healthy food choices.

## Figures and Tables

**Table 1:** Plant-based food product categorization.

Plant-Based Categories		Sub-Categories	Typology
1. Meat Alternatives		Burgers; Sausages; “Meatballs”; Nuggets; Falafel; Tofu and/or seitan; Others (e.g., schnitzels, bites, smoked sausages (plant-based versions of Portuguese traditional smoked sausages such as “Alheira”, “Morcela” and “Chouriço”)).	
2. Dairy Alternatives	Plant-based Beverages	Almond; Oat; Rice; Soy; Blends (contain 2 or more plant-based ingredients); Others (e.g., coconut, hazelnut, tiger-nut, chickpea, pea, cashew).	Plain; Flavored (e.g., vanilla, chocolate)
	Plant-based Yogurts	Soy; Almond; Coconut; Mixed (2 plant-based ingredients)	Natural; Flavored; Greek; Protein
	Plant-based Cheeses	Almond; Coconut oil; Mixed (2 plant-based ingredients)	Spreadable; Slices; Grated
3. Others	Plant-based Ready Meals	Frozen; Canned; Fresh/refrigerated	
	Plant-based Desserts	Creamy; Ice Cream	

**Table 2:** Nutritional composition of plant-based food products categories.

	Number of Products	Energy	Total Fat	Saturated Fat	Total Carbohydrates	Sugars	Fiber	Protein	Salt
Plant-Based Categories		Kcal/100 g	g/100 g	g/100 g	g/100 g	g/100 g	g/100 g	g/100 g	g/100 g
	n (%)	P50 (P25–P75)	P50 (P25–P75)	P50 (P25–P75)	P50 (P25–P75)	P50 (P25–P75)	P50 (P25–P75)	P50 (P25–P75)	P50 (P25–P75)
Meat Alternatives	122 (30)	198 (166–226)	9.2 (7.2–13.0)	1.0 (0.8–1.6)	11.4 (3.2–20.0)	1.5 (0.6–2.7)	3.6 (2.1–6.0)	14.0 (8.8–17.4)	1.2 (0.9–1.5)
Dairy Alternatives	225 (55)	51 (39–64)	1.7 (1.4–2.2)	0.3 (0.2–0.4)	6.8 (2.8–8.5)	4.4 (2.1–6.3)	1.2 (0.4–1.0)	0.8 (0.5–3.3)	0.1 (0.1–0.2)
Others (Ready meals and Desserts)	60 (15)	100 (87–178)	3.2 (2.3–8.1)	0.6 (0.3–3.9)	13.0 (8.1–17.9)	3.4 (2.3–14.8)	2.7 (1.4–3.6)	3.2 (1.2–4.1)	0.4 (0.1–0.7)

**Table 3:** Nutritional composition of plant-based meat alternatives.

		Number of Products	Energy	Total Fat	Saturated Fat	Total Carbohydrates	Sugars	Fiber	Protein	Salt
			Kcal/100g	g/100 g	g/100 g	g/100 g	g/100 g	g/100 g	g/100 g	g/100 g
**Total Meat Alternatives**		n	P50 (P25–P75)	P50 (P25–P75)	P50 (P25–P75)	P50 (P25–P75)	P50 (P25–P75)	P50 (P25–P75)	P50 (P25–P75)	P50 (P25–P75)
		122	198 (166–226)	9.2 (7.2–13.0)	1.0 (0.8–1.6)	11.4 (3.2–20.0)	1.5 (0.6–2.7)	3.6 (2.1–6.0)	14.0 (8.8–17.4)	1.2 (0.9–1.5)
Categories	Burgers	37	206 (179–230) ^a^	8.9 (5.2–12.0) ^bc^	1.0 (0.8–1.3) ^b^	12.0 (7.7–19.0) ^b^	2.2 (1.3–3.1) ^abc^	3.7 (2.6–6.2) ^b^	14.1 (12.0–18.0) ^a^	1.3 (1.0–1.8) ^bc^
	Sausages	12	224 (224–224) ^a^	18.0 (18.0–18.0) ^a^	2.4 (2.3–2.4) ^a^	0.6 (0.6–3.5) ^d^	0.5 (0.5–0.5) ^e^	0.5 (0.5–0.7) ^d^	15.0 (12.0–15.0) ^a^	2.0 (1.5–2.0) ^a^
	Meatballs	8	196 (173–224) ^a^	8.1 (6.8–14.6) ^b^ ^c^	1.1 (0.8–1.8) ^b^	10.0 (4.8–16.1) ^c^	1.1 (0.9–3.0) ^c^	4.2 (2.6–4.9) ^ab^	16.0 (9.3–19.0) ^a^	1.4 (1.1–1.6) ^bc^
	Nuggets	6	250 (211–250) ^a^	13.5 (9.6–17.0) ^b^	1.1 (0.9–1.4) ^b^	20.0 (15.0–24.0) ^ab^	0.6 (0.5–0.8) ^d^	4.8 (3.6–5.9) ^ab^	11.6 (9.7–33.4) ^a^	1.2 (1.0–1.3) ^bc^
	Falafel	10	194 (190–232) ^a^	10.0 (7.6–12.0) ^bc^	1.0 (0.8–1.0) ^b^	19.5 (18.5–22.0) ^ab^	2.8 (2.0–4.3) ^abc^	5.9 (4.6–9.5) ^a^	6.7 (5.9–7.4) ^b^	1.1 (0.9–1.2) ^c^
	Tofu and Seitan	19	146 (125–150) ^b^	8.1 (2.2–9.1) ^ c ^	1.4 (0.7–1.8) ^b^	0.9 (0.1–3.4) ^d^	0.5 (0.1–1.2) ^de^	1.4 (0.8–2.0) ^ c ^	16.5 (14.0–22.1) ^a^	0.1 (0.0–0.2) ^d^
	Others	30	183 (149–236) ^a^	8.2 (5.4–13.0) ^bc^	0.8 (0.6–1.5) ^b^	17.3 (3.1–22.2) ^abc^	1.8 (1.1–2.4) ^bc^	4.5 (2.7–6.3) ^ab^	13.2 (5.6–17.2) ^a^	1.0 (0.9–1.4) ^ c ^
		*p*-value	<0.001	<0.001	0.006	<0.001	<0.001	0.001	<0.001	<0.001
Brand	Private	12	230 (159–304)	6.2 (2.2–11.8)	1.1 (0.5–2.3)	22.4 (12.5–40.5)	2.6 (1.0–6.2)	4.6 (1.4–9.1)	11.7 (8.9–13.5)	1.4 (0.8–3.0)
	Industrial	110	194 (166–225)	9.2 (7.4–13.0)	1.0 (0.8–1.6)	9.9 (3.1–19.0)	1.4 (0.5–2.6)	3.7 (2.2–5.9)	14.4 (8.8–17.6)	1.2 (0.9–1.5)
		*p*-value	0.262	0.061	0.685	0.006	0.048	0.548	0.159	0.506

^a,b,c,d,e^ homogenous subsets according to the Mann–Whitney test with 95% confidence.

**Table 4:** Nutritional composition of plant-based beverages.

		Number of Products	Energy	Total Fat	Saturated Fat	Total Carbohydrates	Sugars	Fiber	Protein	Salt
			Kcal/100 mL	g/100 mL	g/100 mL	g/100 mL	g/100 mL	g/100 mL	g/100 mL	g/100 mL
**Total Plant-based Beverages**		n	P50 (P25–P75)	P50 (P25–P75)	P50 (P25–P75)	P50 (P25–P75)	P50 (P25–P75)	P50 (P25–P75)	P50 (P25–P75)	P50 (P25–P75)
		145	46 (32–56)	1.5 (1.1–1.8)	0.2 (0.1–0.3)	6.6 (2.7–8.4)	4.0 (1.9–5.3)	0.5 (0.4–0.9)	0.9 (0.5–2.1)	0.1 (0.08–0.13)
Categories	Almond	24	26 (18–35) ^b^	1.4 (1.2–1.7) ^b^	0.1 (0.1–0.2) ^c^	3.0 (0.4–3.4) ^c^	3.0 (0.0–3.2) ^bcd^	0.4 (0.3–0.6) ^b^	0.6 (0.5–0.7) ^cd^	0.12 (0.09–0.14)
	Oat	43	48 (43–56) ^a^	1.5 (0.8–1.6) ^b^	0.2 (0.1–0.3) ^b^	7.7 (7.0–8.9) ^b^	4.5 (4.0–6.1) ^a^	0.8 (0.5–1.2) ^a^	0.9 (0.6–1.2) ^bcd^	0.1 (0.09–0.1)
	Rice	14	54 (46–57) ^a^	1.0 (0.8–1.0) ^c^	0.1 (0.1–0.1) ^c^	11.0 (8.3–12.0) ^a^	4.8 (4.6–6.5) ^a^	0.3 (0.0–0.5) ^c^	0.2 (0.1–0.4) ^e^	0.1 (0.09–0.1)
	Soy	35	46 (36–57) ^a^	1.8 (1.6–1.9) ^a^	0.3 (0.3–0.3) ^a^	4.5 (1.8–5.4) ^c^	2.7 (1.7–5.1) ^cd^	0.6 (0.4–0.9) ^ab^	3.0 (3.0–3.2) ^a^	0.1 (0.09–0.14)
	Blend	17	45 (30–55) ^a^	1.4 (1.2–2.2) ^b^	0.3 (0.2–0.3) ^ab^	6.7 (3.4–9.4) ^b^	4.3 (2.2–5.8) ^abc^	0.6 (0.4–0.8) ^ab^	0.5 (0.4–1.2) ^cd^	0.1 (0.08–0.1)
	Others	12	33 (23–48) ^b^	1.4 (1.3–1.8) ^b^	0.3 (0.2–1.0) ^ab^	2.5 (1.0–7.4) ^c^	2.1 (0.0–3.6) ^d^	0.5 (0.2–1.2) ^ab^	0.4 (0.4–2.2) ^d^	0.1 (0.08–0.14)
		*p*-value	<0.001	<0.001	<0.001	<0.001	<0.001	<0.001	<0.001	0.404
Type	Plain	121	42 (30–51)	1.4 (1.1–1.7)	0.2 (0.1–0.3)	5.7 (2.5–8.3)	3.3 (1.5–4.6)	0.5 (0.3–0.8)	0.7 (0.4–1.5)	0.1 (0.08–0.1)
	Flavored	24	57 (50–62)	1.7 (1.5–1.9)	0.3 (0.3–0.4)	7.7 (5.4–9.6)	6.0 (4.8–7.0)	0.9 (0.5–1.1)	2.6 (1.3–3.1)	0.14 (0.1–0.17)
		*p*-value	<0.001	0.002	<0.001	0.034	<0.001	0.004	<0.001	<0.001
Brand	Private	26	46 (34–54)	1.6 (1.2–1.9)	0.2 (0.1–0.3)	7.3 (3.2–8.4)	4.5 (2.7–6.0)	0.5 (0.3–1.2)	1.1 (0.5–2.5)	0.1 (0.08–0.1)
	Industrial	119	46 (31–56)	1.5 (1.1–1.8)	0.2 (0.1–0.3)	6.0 (2.6–8.5)	3.8 (1.8–5.0)	0.5 (0.4–0.9)	0.9 (0.5–2.1)	0.1 (0.09–0.14)
		*p*-value	0.797	0.797	0.284	0.987	0.738	0.225	0.178	0.596

^a,b,c,d,e^ homogenous subsets according to the Mann–Whitney test with 95% confidence.

**Table 5:** Nutritional composition of plant-based yogurts.

		Number of Products	Energy	Total Fat	Saturated Fat	Total Carbohydrates	Sugars	Fiber	Protein	Salt
			Kcal/100 g	g/100 g	g/100 g	g/100 g	g/100 g	g/100 g	g/100 g	g/100 g
**Total Plant-based Yogurts**		n	P50 (P25–P75)	P50 (P25–P75)	P50 (P25–P75)	P50 (P25–P75)	P50 (P25–P75)	P50 (P25–P75)	P50 (P25–P75)	P50 (P25–P75)
		73	69 (58–76)	2.0 (2.0–3.0)	0.4 (0.3–1.1)	8.1 (2.6–9.7)	6.8 (2.2–8.2)	0.9 (0.8–1.1)	3.6 (3.2–3.9)	0.18 (0.1–0.24)
Categories	Soy	54	68 (58–74) ^bc^	2.1 (2.0–2.3) ^c^	0.4 (0.3–0.4) ^c^	8.0 (2.6–8.8) ^b^	7.7 (2.2–8.5)	1.0 (0.8–1.1) ^b^	3.7 (3.6–3.9)	0.2 (0.1–0.2) ^a^
	Almond	3	77 (68–83) ^abc^	4.7 (4.1–5.0) ^a^	0.4 (0.4–0.4) ^c^	4.2 (3.9–6.4) ^b^	0.8 (0.4–2.9)	4.5 (4.5–4.5) ^a^	2.0 (1.6–2.1)	0.02 (0.02–0.03) ^c^
	Coconut	12	90 (73–95) ^a^	4.5 (2.8–4.9) ^ab^	3.7 (2.3–4.6) ^a^	11.8 (11.0–12.1) ^a^	6.7 (5.5–8.8)	0.6 (0.1–1.1) ^b^	0.5 (0.5–0.5)	0.07 (0.07–0.09) ^b^
	Mixed	4	56 (53–70) ^c^	3.0 (2.5–3.0) ^b^	1.1 (0.7–1.2) ^b^	2.4 (1.5–7.2) ^b^	2.4 (1.2–5.0)	1.0 (0.8–1.4) ^b^	3.9 (3.8–3.9)	0.17 (0.1–0.3) ^a^
		*p*-value	0.002	<0.001	<0.001	0.013	0.139	<0.001	0.198	<0.001
Type	Natural	15	55 (51–58) ^b^	2.8 (2.3–3.4) ^ab^	0.4 (0.4–1.1)	2.4 (2.1–3.5) ^b^	1.4 (0.0–2.2) ^b^	1.0 (0.9–1.0) ^bc^	3.9 (1.1–4.0)	0.24 (0.07–0.25)
	Flavored	53	69 (63–76) ^a^	2.1 (2.0–2.8) ^b^	0.4 (0.3–0.4)	8.3 (6.1–11.0) ^a^	7.9 (5.6–8.8) ^a^	0.9 (0.8–1.1) ^c^	3.6 (3.2–3.7)	0.16 (0.1–0.23)
	Greek	3	84 (72–85) ^a^	2.7 (2.4–3.0) ^ab^	0.4 (0.4–0.5)	10.5 (6.0–11.3) ^a^	7.5 (3.8–8.6) ^a^	1.8 (1.8–1.8) ^a^	4.6 (4.1–5.2)	0.1 (0.09–0.1)
	Protein	2	76 (68–84) ^a^	3.2 (3.0–3.3) ^a^	0.5 (0.5–0.6)	5.6 (2.6–8.5) ^ab^	5.4 (2.5–8.2) ^a^	1.4 (1.2–1.5) ^b^	5.5 (5.2–5.8)	0.3 (0.22–0.36)
		*p*-value	0.007	0.005	0.171	<0.001	<0.001	0.006	0.083	0.339
Brand	Private	8	76 (70–80)	2.2 (1.8–3.0)	0.35 (0.3–0.4)	10.2 (6.6–12.6)	9.1 (5.1–10.4)	0.5 (0.5–1.0)	3.2 (3.1–3.6)	0.1 (0.08–0.1)
	Industrial	65	68 (57–75)	2.2 (2.0–3.0)	0.4 (0.4–1.1)	8.1 (2.5–9.6)	6.7 (2.2–8.1)	1.0 (0.8–1.1)	3.7 (3.6–3.9)	0.22 (0.11–0.24)
		*p*-value	0.293	0.232	0.034	0.060	0.047	0.092	0.294	0.132

^a,b,c^ homogenous subsets according to the Mann–Whitney test with 95% confidence.

**Table 6:** Nutritional composition of plant-based cheeses.

		Number of Products	Energy	Total Fat	Saturated Fat	Total Carbohydrates	Sugars	Protein	Salt
			Kcal/100 g	g/100 g	g/100 g	g/100 g	g/100 g	g/100 g	g/100 g
**Total Plant-based Cheeses**		n	P50 (P25–P75)	P50 (P25–P75)	P50 (P25–P75)	P50 (P25–P75)	P50 (P25–P75)	P50 (P25–P75)	P50 (P25–P75)
		7	281 (260–292)	23.0 (20.0–24)	21.0 (15.5–21.5)	20.0 (9.5–22.0)	0.0 (0.0–0.24)	0.0 (0.0–1.6)	1.7 (1.5–1.9)
Type	Spreadable	3	239 (167–272)	23.0 (20.2–26.0)	21.0 (15.6–23.5)	8.0 (5.5–9.5)	0.0 (0.0–0.24)	0.0 (0.0–1.4)	1.2 (0.8–1.5)
	Slices	2	283 (282–284)	21.5 (20.8–22.3)	19.5 (18.8–20.3)	22.5 (21.3–23.8)	0.2 (0.1–0.4)	0.2 (0.1–0.4)	2.1 (1.9–2.2)
	Grated	2	290 (285–294)	22.0 (21.0–23.0)	17.5 (15.2–19.8)	22.0 (21.5–22.5)	0.0 (0.0–0.0)	1.5 (0.8–2.2)	2.0 (2.0–2.0)
		*p*-value	0.754	0.924	0.978	0.105	0.577	0.813	0.123

**Table 7:** Nutritional composition of plant-based ready meals and desserts.

		Number of Products	Energy	Total Fat	Saturated Fat	Total Carbohydrates	Sugars	Fiber	Protein	Salt
			Kcal/100 g	g/100 g	g/100 g	g/100 g	g/100 g	g/100 g	g/100 g	g/100 g
**Total Plant-based Ready Meals**		n	P50 (P25–P75)	P50 (P25–P75)	P50 (P25–P75)	P50 (P25–P75)	P50 (P25–P75)	P50 (P25–P75)	P50 (P25–P75)	P50 (P25–P75)
		24	92 (75–136)	3.0 (2.3–5.1)	0.4 (0.3–0.6)	8.3 (5.3–12.7)	2.3 (1.7–2.8)	2.8 (2.2–3.6)	4.0 (3.3–5.1)	0.7 (0.5–1.0)
Categories	Frozen	18	92 (75–135)	2.7 (2.3–3.5)	0.4 (0.2–0.5) ^c^	10.0 (5.9–12.4) ^b^	2.3 (1.6–2.5)	3.2 (2.7–3.8)	4.0 (2.9–4.7) ^ab^	0.7 (0.4–0.8) ^b^
	Canned	3	76 (73–76)	3.7 (3.0–3.7)	0.6 (0.5–0.6) ^b^	4.0 (4.0–6.2) ^b^	3.1 (2.9–3.1)	1.8 (1.8–1.9)	5.7 (4.4–5.7) ^a^	1.0 (1.0–1.1) ^a^
	Refrigerated	2	161 (152–169)	8.9 (6.7–11.0)	2.4 (0.8–3.9) ^a^	16.0 (13.0–19.0) ^a^	1.8 (1.2–2.4)	1.1 (1.0–1.1)	3.6 (3.4–3.9) ^b^	0.9 (0.7–1.1) ^ab^
		*p*-value	0.057	0.091	0.020	0.030	0.099	0.705	0.003	0.040
Brand	Private	6	100 (43–105)	2.9 (2.6–3.5)	0.4 (0.4–0.4)	12.1 (3.6–12.4)	1.6 (1.5–1.8)	3.8 (3.4–4.0)	4.2 (2.3–4.7)	0.4 (0.2–0.8)
	Industrial	18	88 (75–152)	3.3 (2.3–6.7)	0.4 (0.2–0.6)	8.1 (5.9–13.0)	2.4 (2.3–2.9)	2.7 (2.0–3.0)	3.9 (3.4–5.7)	0.7 (0.7–1.0)
		*p*-value	0.537	0.626	0.721	0.770	0.018	0.030	0.713	0.022
Total Plant-based Desserts		36	162 (95–264)	4.3 (2.3–10.0)	3.9 (0.6–8.6)	17.0 (14.0–31.0)	15.0 (11.0–24.1)	1.7 (0.8–3.5)	1.2 (0.5–3.3)	0.14 (0.1–0.4)
Categories	Creamy	17	95 (88–244)	2.3 (1.2–2.9)	0.6 (0.2–2.3)	14.2 (12.9–54.0)	11 (8.8–49.4)	1.5 (0.8–4.1)	1.1 (0.5–3.1)	0.1 (0.1–0.4)
	Ice Cream	19	250 (196–265)	13.9 (10.8–16.0)	11.0 (9.2–12.1)	29.2 (19.6–31.1)	21.2 (17.4–24.9)	1.1 (0.9–1.7)	1.5 (1.2–3.7)	0.3 (0.1–0.4)
		*p*-value	0.002	<0.001	<0.001	0.021	0.009	0.479	0.004	0.382
Brand	Private	12	249 (164–379)	9.4 (1.3–13.5)	7.6 (0.4–11.2)	30.1 (16.8–63.6)	22.6 (15.2–57.1)	2.3 (1.1–3.8)	1.1 (0.5–1.8)	0.2 (0.1–0.4)
	Industrial	24	95 (87–97)	2.9 (2.5–4.3)	2.3 (0.8–3.9)	14.0 (12.0–14.7)	9.0 (6.8–11.0)	1.5 (0.7–3.1)	1.5 (0.5–3.2)	0.1 (0.1–0.1)
		*p*-value	0.018	0.856	0.830	0.078	0.006	0.464	0.265	0.097

^a,b,c^ homogenous subsets according to the Mann–Whitney test with 95% confidence.

**Table 8:** Nutritional profile and degree of processing of plant-based food products by categories.

		Total Plant-Based Products	Meat Alternatives	Dairy Alternatives			Others	
				Beverages	Yogurts	Cheeses	Ready Meals	Desserts
		n (%)	n (%)	n (%)	n (%)	n (%)	n (%)	n (%)
**Total Fat**	LOW (≤3 g)	238 (58.5)	15 (12.3)	84 (57.9)	57 (78.1)	0 (0.0)	12 (50.0)	12 (33.3)
	MEDIUM (3–17.5 g)	145 (35.6)	94 (77.0)	61 (42.1)	16 (21.9)	1 (14.3)	12 (50.0)	19 (52.8)
	HIGH (≥17.5 g)	24 (5.9)	13 (10.7)	0 (0.0)	0 (0.0)	6 (85.7)	0 (0.0)	5 (13.9)
**Saturated Fat**	LOW (≤1.5 g)	323 (79.4)	85 (69.7)	135 (93.1)	61 (83.6)	0 (0.0)	22 (91.7)	10 (27.8)
	MEDIUM (1.5–5 g)	54 (13.3)	34 (27.9)	10 (6.9)	10 (13.7)	0 (0.0)	2 (8.3)	8 (22.2)
	HIGH (≥5 g)	30 (7.4)	3 (2.4)	0 (0.0)	2 (2.7)	7 (100)	0 (0.0)	18 (50.0)
**Sugars**	LOW (≤5 g)	280 (68.8)	113 (92.6)	46 (31.7)	28 (38.4)	7 (100)	24 (100)	2 (5.6)
	MEDIUM (5–22.5 g)	114 (28.0)	9 (7.4)	99 (68.3)	45 (61.6)	0 (0.0)	0 (0.0)	21 (58.3)
	HIGH (≥22.5 g)	13 (3.2)	0 (0.0)	0 (0.0)	0 (0.0)	0 (0.0)	0 (0.0)	13 (36.1)
**Salt**	LOW (≤0.3 g)	258 (63.5)	14 (11.5)	144 (99.3)	69 (94.5)	0 (0.0)	2 (8.3)	30 (83.3)
	MEDIUM (0.3–1.5 g)	106 (26.1)	72 (59.02)	1 (0.7)	4 (5.5)	2 (28.6)	21 (87.5)	6 (16.7)
	HIGH (≥1.5 g)	42 (10.3)	36 (29.5)	0 (0.0)	0 (0.0)	5 (71.4)	1 (4.2)	0 (0.0)
**NOVA Classification**	Group 1: no/minimaly processed	1 (0.2)	-	-	-	-	1 (4.2)	-
	Group 3: Processed	63 (15.5)	17 (13.9)	33 (22.8)	3 (4.1)	-	10 (41.7)	-
	Group 4: Ultra-Processed	343 (84.3)	105 (86.1)	112 (77.2)	70 (95.9)	7 (100.0)	13 (54.2)	36 (100.0)

**Table 9:** Total number of ingredients and respective total number of ultra-processed food items that compose the different plant-based food products.

Plant-Based Food Categories			Total nº of Ingredients	*p* Value	Total nº of Ingredients (Group 4—NOVA)	*p* Value
			P50 (P25–P75)		P50 (P25–P75)	
Meat Alternatives		Burgers	16 (13–19)	<0.001	4 (2–5)	<0.001
		Sausages	16 (14–18)		5 (4–5)	
		Meatballs	20 (17–23)		8 (7–9)	
		Nuggets	22 (21–23)		6 (6–7)	
		Falafel	18 (16–21)		2 (2–2)	
		Tofu&Seitan	9 (8–14)		1 (1–1)	
		Others	17 (13–22)		3 (2–5)	
Dairy Alternatives	Beverages	Almond	10 (6–13)	<0.001	3 (3–4)	<0.001
		Oat	8 (5–11)		1 (0–2)	
		Rice	6 (4–10)		1 (0–2)	
		Soy	11 (10–13)		3 (2–4)	
		Blend	11 (8–12)		3 (2–4)	
		Others	10 (5–12)		2 (0–4)	
	Yogurts	Soy	17 (13–20)	>0.05	4 (2–5)	>0.05
		Almond	9 (8–9)		4 (4–4)	
		Coconut	15 (8–17)		4 (4–6)	
		Mixed	17 (14–20)		4 (3–7)	
	Cheeses	Almond	6	>0.05	2	>0.05
		Coconut oil	9 (8–11)		4 (2–5)	
Other	Ready Meals	Frozen	15 (11–25)	>0.05	2 (0–5)	>0.05
		Canned	20 (20–20)		3 (2–3)	
		Refrigerated	12 (7–18)		3 (1–4)	
	Desserts	Creamy	11 (10–13)	<0.001	4 (3–7)	<0.001
		Ice Cream	20 (18–23)		9 (7–10)	
**Total plant-based food products**			15 (10–18)		4 (2–5)	

**Table 10:** Description of the main NOVA group 4 ingredients presented among the plant-based foods categories.

Ultra-Processed Foods		Meat Alternatives	Dairy Alternatives			Ready Meals	Desserts
			Beverages	Yogurt	Cheese		
**Food Substances**	**Sugars**	Maltodextrin; dextrose;	Maltodextrine; dextrose; high-fructose corn syrup;	Fruit juice concentrates; high-fructose corn syrup;		Dextrose; Fruit juice concentrates;	High-fructose corn syrup; maltodextrine; Fruit juice concentrates;
	**Modified Oils**	Palm oil;					Palm oil;
	**Sources of protein**	Soy protein isolate; rehydrated pea protein; textured wheat protein; gluten;	Pea protein; Soy protein isolate	Pea protein;	Pea protein;	Soy protein isolate; textured wheat protein; textured pea protein; gluten;	Rehydrated pea protein
**Food Additives**	**Flavours and flavour enhancers**	Different aromas; monosodium glutamate;	Different aromas	Different aromas	Different aromas(e.g., cheese)	Different aromas	Different aromas
	**Artificial sweeteners**		Sucralose; acesulfame K;				
	**Emulsifiers, thickeners, and gelling agents**	Modified starch; Sodium alginate; xanthan gum; carrageenan;	Sunflower lecithin; gellan gum; locust bean gum;	Modified starch; Agar; Pectin;	Modified starch; Agar;	Modified starc; mono- and diglycerides of fatty acids;	Modified starch; xanthan gum; pectin; guar gum; mono- and diglycerides of fatty acids; carrageenan;
	**Others (e.g., colours, and extracts)**	Potato extract; caramel; paprika;	β-Carotene;	β-Carotene;	Olive leaf extract;β-Carotene;	Malted barley extract; paprika extract; Yeast extract;	Coconut extract; β-Carotene;

## Data Availability

The original contributions presented in the study are included in the article, further inquiries can be directed to the corresponding author.
